# The Impact of COVID-19 Lockdown on Adults with Major Depressive Disorder from Catalonia: A Decentralized Longitudinal Study

**DOI:** 10.3390/ijerph20065161

**Published:** 2023-03-15

**Authors:** Raffaele Lavalle, Elena Condominas, Josep Maria Haro, Iago Giné-Vázquez, Raquel Bailon, Estela Laporta, Ester Garcia, Spyridon Kontaxis, Gemma Riquelme Alacid, Federica Lombardini, Antonio Preti, Maria Teresa Peñarrubia-Maria, Marta Coromina, Belén Arranz, Elisabet Vilella, Elena Rubio-Alacid, RADAR-MDD Spain, Faith Matcham, Femke Lamers, Matthew Hotopf, Brenda W. J. H. Penninx, Peter Annas, Vaibhav Narayan, Sara K. Simblett, Sara Siddi, the RADAR-CNS Consortium

**Affiliations:** 1 Dipartimento di Neuroscienze, Università degli Studi di Torino, 10124 Turin, Italy; 2 Parc Sanitari Sant Joan de Déu, Fundació Sant Joan de Déu, CIBERSAM, Departament de Medicina, Universitat de Barcelona, 08830 Barcelona, Spain; 3 Centro de Investigación Biomédica en Red en Salud Mental, CIBERSAM-Instituto de Salud Carlos III, 28029 Madrid, Spain; 4 Aragón Institute of Engineering Research (I3A), Instituto de Investigación Sanitaria de Aragón (IIS Aragón), University of Zaragoza, 50018 Zaragoza, Spain; 5 Centros de Investigación Biomédica en Red en el área de Bioingeniería, Biomateriales y Nanomedicina (CIBER-BBN), 28029 Madrid, Spain; 6 Microelectrónica y Sistemas Electrónicos, Universidad Autónoma de Barcelona, 08193 Bellaterra, Spain; 7 Health Technology Assessment in Primary Care and Mental Health (PRISMA) Research Group, Parc Sanitari Sant Joan de Deu, Institut de Recerca Sant Joan de Deu, 08830 St Boi de Llobregat, Spain; 8 Unitat de Suport a la Recerca Regió Metropolitana Sud, Fundació Institut Universitari per a la Recerca a l’Atenció Primària de Salut Jordi Gol i Gurina (IDIAPJGol), 08007 Barcelona, Spain; 9 Hospital Universitari Institut Pere Mata, 43206 Reus, Spain; 10 Neuriociències i Salut Mental, Institut d’Investigació Sanitària Pere Virgili-CERCA, 43204 Reus, Spain; 11 Universitat Rovira i Virgili, 43003 Reus, Spain; 12 Department of Psychological Medicine, Institute of Psychiatry, Psychology and Neuroscience, King’s College London, London SE5 8AF, UK; 13 School of Psychology, University of Sussex, East Sussex BN1 9QH, UK; 14 Department of Psychiatry, Amsterdam UMC Location Vrije Universiteit Amsterdam, 1081 HV Amsterdam, The Netherlands; 15 Amsterdam Public Health, Mental Health Program, 1081 BT Amsterdam, The Netherlands; 16 H. Lundbeck A/S, DK-2500 Valby, Denmark; 17 Research and Development Information Technology, Janssen Research & Development, LLC, Titusville, NJ 08560, USA

**Keywords:** SARS-CoV-2, depression, anxiety, lockdown, quarantine, remote measurement technology, decentralized study, Spain

## Abstract

The present study analyzes the effects of each containment phase of the first COVID-19 wave on depression levels in a cohort of 121 adults with a history of major depressive disorder (MDD) from Catalonia recruited from 1 November 2019, to 16 October 2020. This analysis is part of the Remote Assessment of Disease and Relapse-MDD (RADAR-MDD) study. Depression was evaluated with the Patient Health Questionnaire-8 (PHQ-8), and anxiety was evaluated with the Generalized Anxiety Disorder-7 (GAD-7). Depression’s levels were explored across the phases (pre-lockdown, lockdown, and four post-lockdown phases) according to the restrictions of Spanish/Catalan governments. Then, a mixed model was fitted to estimate how depression varied over the phases. A significant rise in depression severity was found during the lockdown and phase 0 (early post-lockdown), compared with the pre-lockdown. Those with low pre-lockdown depression experienced an increase in depression severity during the “new normality”, while those with high pre-lockdown depression decreased compared with the pre-lockdown. These findings suggest that COVID-19 restrictions affected the depression level depending on their pre-lockdown depression severity. Individuals with low levels of depression are more reactive to external stimuli than those with more severe depression, so the lockdown may have worse detrimental effects on them.

## 1. Introduction

In January 2020, the World Health Organization declared a novel severe acute respiratory coronavirus disease caused by SARS-CoV-2, the main etiological factor of the disease called COVID-19, Coronavirus Disease 2019 [1]. Soon governments from different countries imposed stringent restrictions to fight COVID-19 diffusion, such as lockdown or self-isolation [2]. On 11 March 2020, the Government of Catalonia introduced social distancing to fight the spread of COVID-19 [3]. The Spanish Government then established strict lockdown measures the day after the declaration of the State of Alarm, on 14 March 2020 [4]. During this first wave of the pandemic, restrictions were lifted gradually through four phases of the post-lockdown implemented by the Spanish government and supplementary measures of the local Catalan government (Table S1). During phase 0 [5,6] non-essential businesses were opened by appointment, and citizens were allowed to do outdoor physical activities by time slot based on age. With phase 1 [4,7,8,9], meetings with a maximum of 10 people were allowed; only outdoor spaces of bars and restaurants opened, as well as some spaces of culture, museums, and gyms; transfers to a second residence were permitted. Later, during phases 2 and 3 [10,11,12,13,14] time slots were abolished, and bars and restaurants’ openings were extended even to the indoor areas, with limited capacity; shopping centers opened; public transport restarted working at 100%; and the percentage of capacity in cinemas, theaters, museums increased. Finally, these relaxations culminated in a period of “new normality”, as the government named it, from 18 June until 16 October 2020, when the COVID-19 second wave started [15].

The benefits in the control of the infection may have been at the expense of significant psychological impact [16]. In particular, individuals with mental health problems were described as running a higher risk of being further psychologically impacted by the pandemic, compared to the general population [17,18,19]. A previous study, conducted on a Spanish sample, showed that during the first phases of the post-lockdown, people with mental illness suffered more from depression symptoms than healthy controls [20]. As regards patients with Major depressive disorder (MDD), some authors [21] found no significant variations of depression severity in an American sample between before and during the lockdown, while others [22] described an increase in a German population.

However, many of the reported studies lack data referring to the pre-pandemic levels of symptoms [20,23,24,25]. Moreover, many were based on cross-sectional surveys [20,22,26,27,28,29,30]. Although there is a large body of literature on the psychological effects of COVID-19, most of it was conducted in the general population [2,18,23,31,32,33,34,35] or included patients with different types of mental disorders in the same sample [26,27,28,29,30,36,37,38,39,40] or used non-standardized instruments to assess the mental status [36,39] or did not take into account the different kinds of restrictions across countries during the first wave of COVID-19 [41].

To adequately understand the impact of the pandemic on depression severity, we need longitudinal studies able to include people with a recent history of MDD. Two previous publications [41,42], using data from the Remote Assessment of Disease and Relapse-Major Depressive Disorder (RADAR-MDD) study [43], explored the depression severity pre-, during, and post-lockdown periods in people with a history of MDD from Spain, the UK, and the Netherlands and observed that patients who displayed significant depression severity shortly before the COVID-19 outbreak decreased in their severity between pre- and during the lockdown [41]. However, both articles [41,42] only considered three periods: pre-lockdown, lockdown, and post-lockdown, but the social restrictions during the post-lockdown were in many places lifted gradually and in different stages, which implies significant differences across countries. The regional and local impact of the COVID-19 crisis has been highly heterogeneous with significant implications for crisis management and policy responses [44]. For example, the Spanish government adopted more restrictive measures than the Netherlands, calculated by the stringency index (highest index per country, which for Spain, the Netherlands, and the UK were 85.2, 78.7, and 79.6, respectively, during the period from February until the first of October 2020) measured by the COVID-19 government response tracker [45]. There is some evidence that policy stringency consisted in physical distancing protocols impeding familiar and meaningful forms of social connection, and more stringent COVID-19 policies were associated with poorer mental health [46].

The present study focuses on one site/country of the RADAR-MDD project to make more detailed analyses on how specific containment measures affected the mental health of individuals with a history of MDD. We aim to explore the impact of the COVID-19 control restrictions on mental health in individuals with a history of MDD in Catalonia (the Spanish sample of the RADAR-MDD study was recruited in this Autonomous Community), adopting a new ‘pandemic-centric’ perspective. RADAR-MDD is part of the RADAR-CNS (Remote Assessment of Disease and Relapse—Central Nervous System) consortium (https://www.radar-cns.org/ accessed on 8 February 2023), which developed the open-source mHealth platform RADAR-Base [47] to collect longitudinal data using remote measurement technology (RMT), using active apps installed in a smartphone providing data on depressed mood, self-esteem, speech, and cognition and passively (without the interactions with the participants) on heart rate, physical activity, sleep, and sociability throughout wearable device and passive app installed in a smartphone. In this study, we assessed the fluctuations of depression and anxiety in individuals with a diagnosis of MDD during the progressive phases of the first wave of the COVID-19 pandemic in relation to the limitations imposed by the national Spanish government and supplementary measures of the local policy of the Catalan authorities. Furthermore, we aimed to verify whether the pandemic influenced the depression levels across phases depending on the pre-lockdown depression and anxiety severity, taking into account the confounding factors. Strong evidence on the specific psychological vulnerability of some individuals to restrictions might contribute to build some new scientific bases, which governments will have to deal with in case of future pandemics.

## 2. Materials and Methods

### 2.1. Study Design and Participants

The present study is based on data gathered in the RADAR-MDD project, refs [43,48] a multi-center cohort study (Netherlands, Spain, and the UK) (https://www.radar-cns.org/ accessed on 8 February 2023) including people with a history of MDD who were evaluated from November 2017 to March 2021. All participants fulfilled the following inclusion and none of the exclusion criteria. They had at least one MDD episode within the last 2 years and at least 2 lifetime episodes of MDD and were over age 18 only. Furthermore, they were able to complete self-reported assessment via smartphone. Exclusion criteria: dementia, pregnancy, major medical disease, an alcohol or drug dependency within the past 6 months, or lifetime history of other mental disorders (bipolar, personality, schizophrenia, MDD with psychotic features, or schizoaffective disorder).

The study was co-developed with service users in our Patient Advisory Board (PAB), who were involved in the choice of measures, the timing, and issues of engagement and have also been involved in developing the analysis plan, and a representative is an author of this paper and critically reviewed it.

In this manuscript, we focused our analyses on the 155 participants with a history of MDD according to the DSM-5 criteria evaluated by clinicians from Catalonia and analyzed the data during the first wave of COVID-19, between 1 November 2019, and 16 October 2020. Of those 155 participants, 22 were not recorded during the periods of interest (from 1 November 2019 to 16 October 2020), and 12 missed records during the pre-lockdown period (2 participants died of COVID-19 infection).

Thus, the final sample included 121 participants.

Additionally, some participants had missing values that referred to certain phases (see the supplementary materials for the number of observations in each phase, Table S2). No differences in the PHQ-8 pre-lockdown values of these patients with missing records in certain phases were observed (*p* > 0.05) as compared to those without any missing data.

#### Pandemic Phases

**Figure 1:**
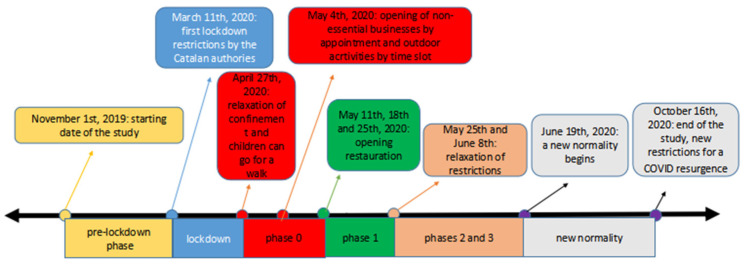
Description of the pre-lockdown and post-lockdown phases.

The phases and restrictions had a longer duration or were applied later depending on the location (Barcelona, Tarragona, or Garraf). The dates of the restrictions are shown in Figure 1, and for a full description, see the supplementary materials (Table S1). On this basis, we focused on the following phases:

▪ Pre-lockdown (from 1 November 2019 to 10 March 2020);
▪ Lockdown (from 11 March 2020 to 26 April 2020);
▪ Later restrictions were lifted gradually through four phases of the post-lockdown:
▪ Phase 0 (from 27 April 2020 to 10 or 17 or 24 May 2020 *);
▪ Phase 1 (from 11 or 18 or 25 May 2020 to 24 May or 7 June 2020 *);
▪ Phases 2 and 3 (from 25 May or 8 June 2020 to 18 June 2020 *);
▪ “New-normality” (from 19 June 2020 to 16 October 2020).

* Depending on the locations

## 3. Measures

### 3.1. Depression

Depression severity was assessed through the PHQ-8 (Patient Health Questionnaire-8) [49]. Participants were required to answer this questionnaire every two weeks. The total score could range from 0 to 24, with increasing number meaning higher severity; a score of 10 was set as a cut-off [49], above which the severity of symptoms could be considered clinically relevant. Internal consistency was calculated on 3310 observations, with Cronbach’s alpha of 0.917.

### 3.2. Anxiety Symptoms

Severity of anxiety was assessed using the Generalized Anxiety Disorder-7 item (GAD-7) scale [50], which was collected at baseline and every 3 months. The GAD-7 score could vary from 0 to 21, with increasing number meaning higher severity, and a score of 10 was assumed as a cut-off [50], above which the severity of symptoms could be considered clinically relevant. Internal consistency was calculated on 1178 observations, with Cronbach’s alpha of 0.971.

### 3.3. Socialdemographic Variables

Gender, comorbidity with medical conditions, age, marital status, number of people living with, employment, income, and age of finishing education were also collected at baseline.

## 4. Statistical Analysis

First, we described the sociodemographic characteristics of the sample and computed a descriptive analysis of the PHQ-8 values among the different lockdown phases. We then calculated each participant’s baseline depression and anxiety severity by taking the average of the PHQ-8 and GAD measures in the pre-lockdown period. Participants were grouped based on their pre-lockdown depression (PHQ-8 ≥ 10 or <10) and their pre-lockdown anxiety levels (GAD-7 ≥ 10 or <10). We analyzed the variations of PHQ-8 in the different phases of the pandemic using the paired Wilcoxon text. In those participants with more than one PHQ-8 evaluation in the same phase, we computed the mean values and used them for the following analyses. We also applied a linear mixed model to analyze the course of depression levels over phases. To identify the covariate variables (sociodemographic variables) that would be included in the linear mixed model, we computed the forward stepwise method implemented with R based on the best Akaike information criterion (AIC) value. We also evaluated the effect size on pre-lockdown depression levels of each variable and added to the model those not selected by the stepwise procedure but with at least a medium effect (Cohen’s d ≥ 0.5 for categorical variables and Spearman correlation coefficient ≥ 0.3 for continuous variables). To investigate how the pre-lockdown depression levels associated with variations in depression severity across phases, the model was stratified by the pre-lockdown depression levels (PHQ-8 cutoff ≥ 10). Random effects of participants were incorporated as well, and the model was adjusted using the maximum likelihood (ML) method, which provided correct estimation when data were not completely missing at random [41]. All statistical analyses were conducted using the nlme package in R [51] and STATA 13.

## 5. Results

Most of the participants were females (66.9%); the median age was 58 years (IQR 52–64) (Table 1). More than half of the individuals had high anxiety (64.5%) and high depression (PHQ-8) (67.8%) levels during the pre-lockdown phase.

**Figure 2:**
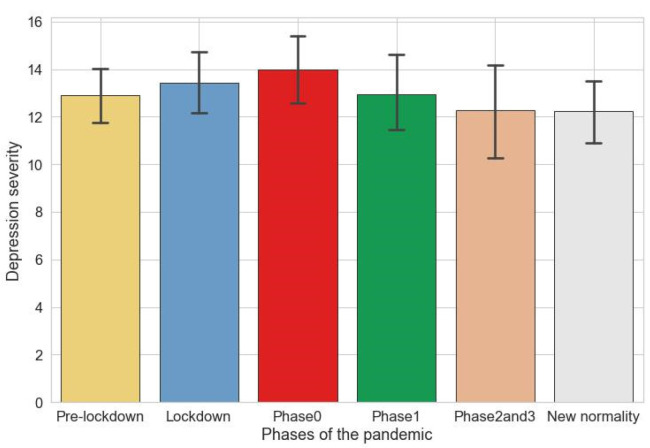
Bar plot of PHQ8 score during the pandemic phases. Each bar corresponds to the mean and confidence intervals of depression severity assessed by PHQ-8.

Figure 2 shows descriptive analysis of the depression levels measured by PHQ-8 values in the different phases of the pandemic.

Using the paired Wilcoxon test, a statistically significant increase in the depression levels (PHQ-8 score) was observed in phase 0 compared to the pre-lockdown (*p* ≤ 0.05) and new-normality (*p* ≤ 0.01) phases, while a significant decrease was found in new-normality as compared with lockdown (*p* ≤ 0.05).

A linear mixed model (Table 2) was fitted to explore the association between phases and anxiety and depression severity, adjusted for the covariates (gender, age, income, number of people living with, and comorbidity with other medical conditions). A significant rise in depression severity was found during the lockdown (*p* ≤ 0.001) and phase 0 (*p* ≤ 0.001), as compared with the pre-lockdown phase. Depression severity was higher in patients with high pre-lockdown anxiety (GAD-7 ≥ 10) (β = 7.955, CI 95% [6.240–9.670], *p* ≤ 0.001) than in those with low pre-lockdown anxiety symptoms (GAD-7 < 10) over all phases. Figure S1 (see Supplementary Materials) shows the depression severity across all phases by pre-lockdown anxiety level. Only the social status measured by the number of people living with (two or more people living with the participant) had a reduced significant effect on depression severity. Indeed, the fact of living with two (β = −2.368, CI 95% [−4.402–−0.334], *p* ≤ 0.022), three (β = −2.494, CI 95% [−4.536–−0.451], *p* ≤ 0.017), or four or more (β = −3.687, CI 95% [−5.923–−1.451], *p* ≤ 0.001) people was found to decrease the depression severity. No significant effects were found for what concerns the other covariates.

**Figure 3:**
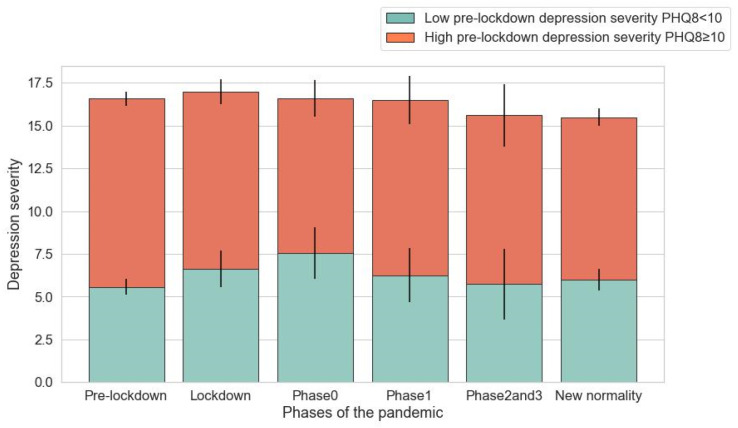
Mean levels and confidence interval of depression (PHQ-8) by pre-lockdown depression severity during each phase. Based on a descriptive analysis, we divided the participants depending on the pre-lockdown depression severity.

**Figure 4:**
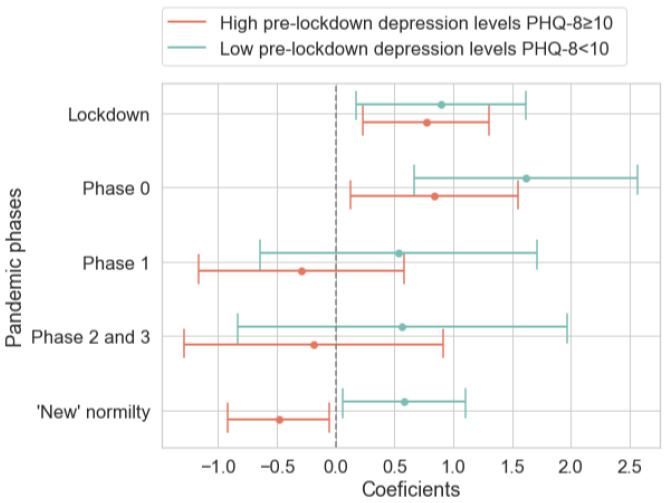
Coefficients of stratification model. Adjusted for the covariates: gender, age, income, number of people living with, and comorbidity with other medical conditions.

We then stratified participants into two groups: one contained subjects with high pre-lockdown depression (82 participants and 1048 observations); the other included subjects with low pre-lockdown depression (39 participants and 547 observations). In this way, we aimed to verify whether the association between the phases and depression severity depended on the pre-lockdown depression severity (see Figure 3 and Figure 4).

According to the first model (Figure 4), participants displayed a significant increase in depression severity during the lockdown (β = 0.768, CI 95% [0.227–1.309], *p* ≤ 0.001) and phase 0 (β = 0.838, CI 95% [0.127–1.550], *p* = 0.021), as compared to the pre-lockdown levels. Moreover, we found a significant decrease in depression levels during the post-lockdown (β = −0.486, CI 95% [−0.917–−0.055], *p* = 0.027).

As regards the second model (Figure 4), participants also displayed a significant increase in their depression severity during the lockdown (β = 0.894, CI 95% [0.174–1.614], *p* = 0.015) and phase 0 (β = 1.615, CI 95% [0.664–2.565], *p* ≤ 0.001), as compared with the pre-lockdown, consistent with the previous model. Instead, we found a significant increase in depression levels during the post-lockdown (β = 0.581, CI 95% [0.056–1.105], *p* = 0.03). The coefficients of the stratified model adjusted for the covariates are represented in Figure 4.

## 6. Discussion

We observed that the participants with a history of MDD were mainly affected during the lockdown and the early phase of post-lockdown, when their depression severity increased as compared to pre-lockdown levels. These findings should not be surprising, since the most stringent measures were imposed during these phases.

In previous literature, some works [20,24] showed that patients with mental illness were more depressed during the post-lockdown phase than healthy controls as expected. Another study [23] observed a predominantly depressive response to lockdown in all the participants with a current or a past mental disorder but also in healthy controls. What is clear is that none of these studies, all of whom conducted in samples of Spanish people and dealt with the specific impact of the pandemic on the depression severity of patients suffering from MDD, unlike two works [21,22] outside Spain which did. Quittkat et al. (2020) showed that in depressed patients from Germany depression severity increased during the lockdown, as compared with the pre-lockdown (November 2019). During this lockdown, strict restrictions were imposed, corresponding, in essence, to those existing in Catalonia during the lockdown, although the lockdown in Spain was more stringent than in Germany. This is clearly shown by the data given by the Oxford COVID-19 government response tracker [45]: an index called stringency level was introduced to describe numerically the severity of restrictions, based on values given to many parameters measuring it, such as the closure of the school, the limitations imposed to internal movements, etc. For what concerns April 2020, Spain had a stringency level of 85.19, whereas Germany had 76.85. In contrast, the study by Hamm et al. [21] in four American metropolitan areas provided evidence contrary to our results, as they did not find significant changes in depression in their cohort with MDD, between pre-lockdown and lockdown. However, this divergence of findings can be explained by a far lower stringency level of restrictions (stringency level [45] of 72.69 in April 2020). Moreover, analyses were conducted on a sample older than ours (the mean age was 69), thus we might also interpret this apparent lesser impact of social restrictions on the elderly due to a weaker habit of going out than younger people.

Furthermore, in these two studies [21,22] patients were submitted single-time inquiries, the periods in analysis corresponded to about a month, and in the former [22] data referred to pre-lockdown. Instead, the present decentralized study, as well as the others [41,42,52] from RADAR-MDD, was longitudinal, referring to a period of about a year, thus offering higher methodological standards. However, in our previous study [41] we did not observe increases in depressive severity across phases (the Netherlands, Spain, and the UK). This divergence might depend on the different restrictions implemented across countries (the Netherlands, Spain, and the UK) [52] or different depression severity across sites [41].

Moreover, other studies [41] were “lockdown-centric”, since the period in study was divided into a pre-lockdown phase, lockdown, and a post-lockdown phase including indiscriminately the whole period following the lockdown. Instead, the present manuscript considered the post-lockdown dividing it into different periods based on the gradual ease of restrictions.

Second, the model suggests that in the “new-normality” period those subjects with high pre-lockdown depression experienced a decrease in depression severity.

These results come out as partly divergent from those found in previous studies, but these discrepancies might again rely on the above-discussed differences in the division into phases of our common period of interest. Indeed, Leightley et al. (2021) included in their post-lockdown phase also the period that in the current study was studied as the phase 1 of the post-lockdown, when some social restrictions were still in place. Thus, as we based our analyses on different divisions of time, slight differences in findings are understandable. However, many studies [20,21,22,23,24,40,53,54] dealt with the fluctuation of depression during the pandemic in depressed patients but definitely did not considering the influence of different levels of pre-lockdown depression or anxiety on the subsequent variation of depression severity due to the COVID-19 first wave. Therefore, to the best of our knowledge, our study is the first one that describes in-depth the influence of the pre-pandemic levels of depression and anxiety in the subsequent oscillations in the depression severity of patients with a history of MDD during the different phases of the pandemic. As explained above, the core of our findings suggests not only that the impact of restrictions in terms of depression worsening was significant during the lockdown and the first phase of post-lockdown but also that the pre-lockdown levels of depression severity exerted a possible influence on the course of depression during the last phase of the post-lockdown. Indeed, during this last phase of the post-lockdown a significant increase of depression was found in those with lower pre-existing levels of depression as compared to the pre-lockdown, whereas in those with higher levels our results displayed a decrease in depression. Furthermore, our sample only consisted of Catalan people living under the same restrictions at the same time, which makes our study population unprecedently homogeneous, also from a socio-cultural perspective.

These results provide unprecedented points of reflection, soliciting the proposal of possible explanations. There is no doubt that the COVID-19 pandemic produced a contraction of the habitual interactions of the individual with the stimuli produced by the external world. Thus, we might suppose that the psychological impact of such everyday life upheaval must have been more critical on those patients whose pre-lockdown depressive and anxiety symptoms were not severe to such an extent that their psychological wellbeing could be influenced by relevant changes in the external world. In other words, high levels of depression before the pandemic might have made individuals less responsive to the stress [55] and prevented them from suffering from a persistent increase in depression severity during the periods of social restrictions [56]. High levels of pre-lockdown depression might have paradoxically worked as a protective factor against the negative impact of the pandemic on depressive symptoms. Indeed, severe depression can impair individuals suffering from it to such an extent that not only could potentially positive aspects of reality not affect them and improve their emotional status, but also potentially harmful stimuli, such as lockdowns, might fail to move their psychological condition somehow. Instead, those individuals suffering from low pre-lockdown levels of depression are still emotionally interactive with the external world. Thus, lockdown might have had the “possibility” to impact their symptoms negatively.

However, given the scarcity of past references on the theme, these hypotheses need to be further proved by future analyses on the possible role of pre-pandemic levels of depression and anxiety as predictors for the impact of severe social restrictions on the course of depression severity in patients affected by MDD.

## 7. Implications and Future Directions

First, the study of the effects of restrictions on depression severity in a clinical population of people suffering from MDD must be regarded as providing a relevant clinical implication. The results of the current work about the specific psychological impact of the different measures imposed a long time and could inform authorities in planning a future policy of restrictions in the case of new pandemics, taking into account more accurately and from several points of view the benefits and disadvantages of applying social restrictions.

For example, analyses on our sample suggest that during the lockdown and the early phase of the post-lockdown, when only some outdoor sport activities were allowed, the levels of depression severity remained high, whereas a drop was registered during phase 1, when some restrictions of social activities were slightly eased. Thus, it is hoped that, in case of future pandemics, this specific vulnerability to restrictions that we described in depressed patients will induce authorities to, for example, exempt them from the strictest forms of lockdown, compatibly with the epidemiological context.

In future research, we want to extend our study of the variations of depression levels across phases of pandemics to different waves of the COVID-19 pandemic and to different countries. To analyze this, it will be mandatory to take again into account the specific restrictions imposed across time and different areas.

## 8. Limitations and Strengths

Some limitations must be considered. The assessment of severity was self-reported and may have lower reliability than heteroadministered (administered by a physician on behalf of a patient) questionnaires or tests. Furthermore, some subjects missed data referring to certain phases.

Strengths of the study include a homogeneous sample in terms of geographical origin, and we were able to analyze lockdown measures in detail; however, this limits the generalizability of the findings. Since we considered the differences between different areas within Catalonia, each group of patients corresponding to a specific phase contained only participants who were imposed under the same restrictions at the same time. Moreover, thanks to the length of our period of assessment, for each participant several observations have been available, referring to the pre-lockdown, lockdown, and post-lockdown phases. Thus, the present research went beyond the “lockdown-centric” perspective of the previous literature and has to be considered the first one assuming a “pandemic-centric” point of view, which not only considered the acute impact of the most severe restrictions but also aimed at detecting the subtle variations in depression during the post-lockdown phases.

Furthermore, due to the employment of RMT and the prospectively planned, longitudinal design of our study, recall bias was completely prevented. Also, our database allowed us to distinctly study the role of pre-lockdown levels of depression and anxiety as predictors for variations in depression severity during lockdown and post-lockdown.

## 9. Conclusions

Future studies will have the possibility to refer to the present work as a significant milestone of this new “pandemic-centric” perspective, based on which not only an impairment in depression severity during the first phases of pandemic was found, but also a more severe impact of the pandemic was described on those patients suffering from milder forms of MDD. Thus, our results emphasize the strong need for attentive care that these specific populations run under social restrictions, hopefully contributing to the process of turning the spotlight on the psychological impact of the pandemics.

## Figures and Tables

**Table 1:** Characteristics of the sample.

Variables		N = 121
Gender, N (%)	Male	40 (33.1)
	Female	81 (66.9)
Comorbidity ^1^, N (%)	Yes	74 (61.2)
	No	47 (38.8)
Age, Median (IQR)		58 (52–64)
Marital status, N (%)	with a partner	68 (56.2)
	without a partner	53 (42.8)
People living with, N (%)	alone	22 (18.2)
	two	40 (33.1)
	three	37 (30.6)
	four or more	22 (18.2)
Employment, N (%)	Employed	43 (35.5)
	Unemployed ^2^	78 (64.5)
Income ^3^, N (%)	<15,000 €	33 (27.3)
	15,000 €–24,000 €	48 (39.7)
	>24,000 €	40 (33.1)
Age of finishing education, mean (SD)		17.6 (4.95)
Pre-lockdown PHQ-8	Mean	12.90
	Median (IQR)	13 (10.2)
Pre-lockdown GAD-7	Mean	10.76
	Median (IQR)	10 (5.2)
Pre-lockdown PHQ-8, N (%)	PHQ-8 < 10	39 (32.2)
	PHQ-8 ≥ 10	82 (67.8)
Pre-lockdown GAD-7, N (%)	GAD-7 < 10	43 (35.5)
	GAD-7 ≥ 10	78 (64.5)

^1^ Comorbidity the simultaneous presence of other diseases or medical conditions in a patient apart of the depression. ^2^ Unemployment is also considered retirement or work leave. ^3^ The values of income correspond to the gross salary per year.

**Table 2:** Depression severity across phases compared to pre-lockdown levels measured with the PHQ-8 (Linear mixed model).

	Coef. ^1^	CI (95%)	*p*-Value
Pre-lockdown	Ref	-	-
Lockdown	0.866	[0.430 to 1.303]	≤0.001
Phase 0	1.135	[0.560 to 1.711]	≤0.001
Phase 1	−0.004	[−0.709 to 0.700]	0.990
Phase 2–3	0.098	[−0.770 to 0.967]	0.824
New-normality	−0.099	[−0.437 to 0.238]	0.562

^1^ Mixed model was adjusted for the covariates: gender, age, income, and number of people living with, and comorbidity with other medical conditions.

## Data Availability

The datasets used and or analyzed during the current study are available from the corresponding author on reasonable request.

## Supplementary Materials

The following supporting information can be downloaded at: https://www.mdpi.com/article/10.3390/ijerph20065161/s1, Document S1. Description of the lockdown phases. Table S1: Dates of each phase of the pandemic in Catalonia, based on the three studied districts. Table S2: Number of participants, observations, and average PHQ-8 value in each phase. Figure S1. Mean levels and confidence interval of depression (PHQ-8) by pre-lockdown anxiety severity during each phase. Based on a descriptive analysis, we divided the participants depending on the pre-lockdown anxiety severity (GAD-7). References [57,58,59,60,61,62,63,64] are cited in the Supplementary Materials.

## Abbreviations

aRMT	active remote measurement technology
GAD-7	item Generalized anxiety disorder-7 item
IQR	Interquartile ranges
MDD	Major depressive disorder
ML	maximum likelihood
pRMT	passive remote measurement technology
PHQ-8	Patient health questionnaire 8-item
RADAR-CNS	Remote assessment of disease and relapse—central nervous system
REDCap	Research electronic data capture
RADAR-MDD	Remote Assessment of Disease and Relapse-Major Depressive Disorder
