# Supplementary material for: The Impact of COVID-19 Lockdown on Adults with Major Depressive Disorder from Catalonia: A Decentralized Longitudinal Study
Source: Int J Environ Res Public Health. 2023 Mar 15;20(6):5161. doi: 10.3390/ijerph20065161 (PMC10048808; doi:10.3390/ijerph20065161)
Supplement: Supplementary file 1 [file ijerph-20-05161-s001.zip › ijerph-2213595-supplementary.pdf]

SUPPLEMENTARY MATERIALS

Document S1. Description of the lockdown phases.

Different phases of restrictions in Catalonia, based on the restrictions of the three studied districts:

- Pre-lockdown (from November 1, 2019, to March 11, 2020): for this period, we chose November 1 as the starting date until March 11. Indeed, on this last day, the Government of Catalonia introduced social distancing to fight the spread of COVID-19 [3]
- Lockdown (from March 11, 2020, to April 27, 2020): on March 14, 2020 [57] was declared the State of Alarm, andthe Spanish population was subjected to a severe lockdown from the following day. Mobility was limited to essential activities and educational institutions were closed [58]. Then, the Catalan authorities introduced supplementary measures, on March 12, 13, 18, 23 [59–62], which produced the suspension of any in-person educational activity, the closure of libraries and museums, a temporary reorganization of the health system, etc.

Later restrictions were lifted gradually through four phases of the post-lockdown:

- Phase 0 (from April 27, 2020, to May 10 or 17 or 24, 2020 depending on the district): restrictions began to be lifted on May 4 in all of Spain [5,6], when non-essential businesses were opened by appointment and citizens were allowed to do outdoor physical activities (1h per day maximum) by time slot based on age. In this study, we considered April 27 as the beginning of phase 0, since the relaxation of confinement and the permission for children to go for a walk had already been instituted seven days earlier than May 4
- Phase 1 (from May 11 or 18 or 25 2020 to May 24 or June 7, depending on the district): [4,7,8,10] measures were gradually relaxed: meetings with a maximum of 10 people were allowed; the bars and restaurants opened (only outdoor space), as well as some spaces of culture, museums, and gyms; transfers to a second residence were permitted.
- Phases 2 and 3 (from May 25 or June 8 2020 to June 18 depending on the district): [9,12,13,63] time slots were finished and bars and restaurants' openings were extended even to the indoor areas, with limited capacity. Shopping centers opened, public transport restarted working at 100%, and the percentage of capacity in cinemas,theaters, and museums increased. Phase 3 slightly differed from the previous one: themain discrepancies consisted in a higher capacity allowed for indoor activities and in the fact that ceremonies could again take place. Phases 2 and 3 were considered together, as the latter was very short, for example, 2 days in total in Barcelona [11,14], and differences among them were minimal.
- New normality (from June 19 to October 16): on June 18 the Catalan authorities signed the *resoluciò INT/1433/2020* [15]: finally, the Spanish population could experience a "new normality". The only resisting restrictions concerned social distancing, the use of a mask, avoidance of crowds of people, and maintenance of some capacity limitations. October 16, 2020, was chosen as the end of the phase when there was a resurgence of COVID-19 and thegovernment set in place new restrictions [64].

Table S1. Dates of each phase of the pandemic in Catalonia, based on the three studied districts

Phase of pandemic	Distructs	Starting day	Ending day
Pre- lockdown	BAR, TAR and GAR	01/11/2019	10/03/2020
Lockdown	BAR, TAR and GAR	11/03/2020	26/04/2020
Phase 0	BAR	27/04/2020	24/05/2020
	TAR	27/04/2020	10/05/2020
	GAR	27/04/2020	17/05/2020
Phase1	BAR	25/05/2020	07/06/2020
	TAR	11/05/2020	24/05/2020
	GAR	18/05/2020	07/06/2020
Phase 2 and 3			
	BAR	08/06/2020	18/06/2020
	TAR	25/05/2020	18/06/2020
	GAR	08/06/2020	18/06/2020
New normality	BAR, TAR and GAR	19/06/2020	16/10/2020

***Note**: BAR: Barcelona, TAR: Tarregona, GAR: Garraf.*

Table S2. *Number of participants, observations, and average PHQ-8 values in each*
phase

Phases	Observations	N	PHQ8		
			Mean	CI (95%)	Median
Pre-lockdown	659	121	12.788	[12.28 to 13.29]	13
Lockdown	215	88	13.763	[12.88 to 14.64]	15
Phase 0	109	72	13.688	[12.51 to 14.86]	14
Phase 1	66	63	13.227	[11.64 to 14.81]	14
Phases 2-3	43	40	12.163	[10.18 to 14.15]	12
New normality	503	84	12.048	[11.49 to 12.61]	12

Note: *The observations are the PHQ-8 assessments of each phase. N corresponds to the number of people ofthose 121 who have registration in the corresponding phase.*

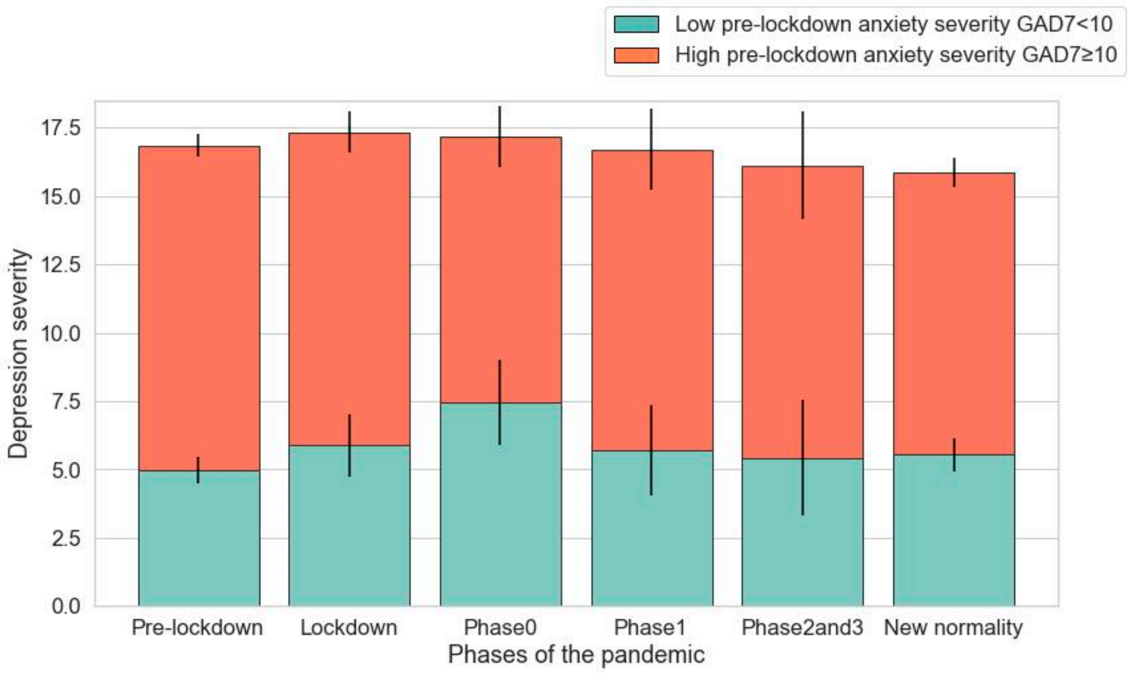

Figure S3. Mean levels and confidence interval of depression (PHQ-8) by pre-lockdown anxiety severity during each phase. *Based on a descriptive analysis, we divided the participants depending on the pre-lockdown anxiety severity (GAD-7).*
